# LXR-Mediated ABCA1 Expression and Function Are Modulated by High Glucose and PRMT2

**DOI:** 10.1371/journal.pone.0135218

**Published:** 2015-08-19

**Authors:** Maryem A. Hussein, Elina Shrestha, Mireille Ouimet, Tessa J. Barrett, Sarah Leone, Kathryn J. Moore, Yann Hérault, Edward A. Fisher, Michael J. Garabedian

**Affiliations:** 1 Department of Microbiology, NYU School of Medicine, New York, New York, United States of America; 2 Department of Medicine, NYU School of Medicine, New York, New York, United States of America; 3 Institut de Génétique et de Biologie Moléculaire et Cellulaire, Illkirch, 1 rue Laurent Fries, 67404, Illkirch, France; 4 Centre National de la Recherche Scientifique, UMR7104, Illkirch, France; 5 Institut National de la Santé et de la Recherche Médicale, U964, Illkirch, France; 6 Université de Strasbourg, Illkirch, France; Institut Clinique de la Souris, ICS, 1 rue Laurent Fries, 67404, Illkirch, France; University of Geneva, SWITZERLAND

## Abstract

High cholesterol and diabetes are major risk factors for atherosclerosis. Regression of atherosclerosis is mediated in part by the Liver X Receptor (LXR) through the induction of genes involved in cholesterol transport and efflux. In the context of diabetes, regression of atherosclerosis is impaired. We proposed that changes in glucose levels modulate LXR-dependent gene expression. Using a mouse macrophage cell line (RAW 264.7) and primary bone marrow derived macrophages (BMDMs) cultured in normal or diabetes relevant high glucose conditions we found that high glucose inhibits the LXR-dependent expression of ATP-binding cassette transporter A1 (ABCA1), but not ABCG1. To probe for this mechanism, we surveyed the expression of a host of chromatin-modifying enzymes and found that Protein Arginine Methyltransferase 2 (PRMT2) was reduced in high compared to normal glucose conditions. Importantly, ABCA1 expression and ABCA1-mediated cholesterol efflux were reduced in *Prmt2* ^-/-^ compared to wild type BMDMs. Monocytes from diabetic mice also showed decreased expression of *Prmt2* compared to non-diabetic counterparts. Thus, PRMT2 represents a glucose-sensitive factor that plays a role in LXR-mediated ABCA1-dependent cholesterol efflux and lends insight to the presence of increased atherosclerosis in diabetic patients.

## Introduction

Diabetes mellitus is characterized by accelerated atherosclerosis and higher risk for cardiovascular diseases [1–4]. Atherosclerosis in diabetic patients is often associated with increased plaque macrophages and dyslipidemia [5]. Though diabetes is a complex multifactorial disease it is notable that high blood glucose levels have been shown to be an independent risk factor for atherosclerosis [1, 6–8]. We and others have established previously in mouse models that lowering cholesterol levels promotes the regression of atherosclerosis [9–11]. This is partly mediated by the Liver X Receptor (LXR), a transcription factor that induces the expression of genes involved in cholesterol transport and efflux, a process known as reverse cholesterol transport [12–14].

LXRs (LXRα and LXRβ) are members of the nuclear receptor family of transcription factors [15]. They form an obligate heterodimer with the retinoid X receptor (RXR) and, in response to either receptor’s ligand, upregulate genes involved in cholesterol transport and efflux [16–18]. In a mouse model of atherosclerosis regression, diabetic mice show less plaque reduction compared to their normoglycemic counterparts despite equivalent serum lipid profiles [19]. We hypothesized that high glucose alters the repertoire of LXR regulated genes in macrophages and predicted that changes in gene transcription and expression would inhibit their ability to efficiently efflux cholesterol under diabetic conditions.

Macrophages respond to cholesterol accumulation by increasing the expression of ATP-binding cassette transporter A1 (ABCA1), to promote the removal of cholesterol and other lipids from the cell [14, 20–22]. ABCA1 is known to transfer cholesterol from macrophages to the specific lipid-poor cholesterol acceptor, apolipoprotein A1 (APOAI) [14, 21]. In macrophages, excess cholesterol leads to the formation of oxysterols, the natural ligands of LXR, which stimulate *Abca1* expression [20]. We found that macrophages cultured under elevated glucose conditions showed decreased LXR-dependent ABCA1 mRNA and protein levels. Importantly, this led to a defect in ABCA1-mediated efflux to APOAI. In an effort to understand the mechanism of this glucose-mediated transcriptional effect, we looked to processes known to modify the chromatin landscape. Gene transcription is a dynamic process involving the conversion of compact heterochromatin into transcription factor accessible euchromatin. Chromatin modifications of different classes (e.g. acetylation, methylation, ubiquitination, phosphorylation) positively or negatively impact gene expression and play an important role in gene regulation [23, 24]. We examined the expression of chromatin modifying enzymes in these cells and found that the level of a protein arginine methyltransferase, PRMT2, was lower in macrophages in a high glucose environment. This was also true in monocytes isolated from diabetic mice that were generated using streptozotocin (STZ), a drug that targets and ablates the insulin-producing pancreatic beta cells [25]. In the absence of insulin, these mice are unable to appropriately absorb sugar from the blood into tissues responsible for sugar metabolism, which leads to excess levels of blood glucose. This mechanism of hyperglycemia simulates the Type 1 diabetic condition. Given that PRMT2 was previously shown to act as a coactivator for other members of the nuclear receptor family, we hypothesized that PRMT2 could serve as a regulator for LXR-dependent gene expression [26, 27]. Using macrophages from mice lacking the PRMT2 gene, we found that the lack of PRMT2 mimicked the effects of high glucose. Macrophages from mice lacking PRMT2 had decreased LXR-mediated upregulation of ABCA1 and a profound defect in ABCA1-mediated cholesterol efflux to APOAI. Thus, PRMT2 represents a glucose-sensitive factor that plays a role in LXR-mediated *Abca1* expression and ABCA1-dependent cholesterol efflux.

## Materials and Methods

### RAW264.7 cells ectopically expressing human LXRα

RAW264.7 cells stably expressing FLAG-tagged human LXRα (herein referred to as RAW WT) previously described in [28] were maintained in Dulbecco's modified Eagle's medium (DMEM, Gibco) with 10% fetal bovine serum (FBS) and 1% PenStrep (100 U/mL Penicillium and 100ug/mL Streptomycin) in either 4.5 g/L D-glucose or 1 g/L D-glucose + 3.5 g/L L-glucose (Sigma) as an osmotic control. Cells were maintained in these glucose conditions for a minimum of two weeks before being used and were cultured in 1% FBS overnight before experiments. Cells were cultured in 5% CO_2_ atmosphere at 37°C. Cells were tested for mycoplasma and were negative.

### Bone marrow derived macrophages (BMDMs)

BMDMs were prepared from monocytes isolated from the tibia and femur of 6–10-week-old C57BL/6 male mice. A total of 18 mice were used to perform the experiments. The animals were maintained with a 12-hour light-dark cycle and had free access to food and water in a pathogen-free facility with no more than 5 mice (< 25 g) per cage. Mice were euthanized by CO_2_ followed by cervical dislocation in accordance with AVMA Guidelines for the Euthanasia of Animals. Bone marrow cells were isolated, treated with red blood cell lysis buffer (Sigma) and re-suspended in differentiation medium (DMEM with 1 g/L D-glucose + 3.5 g/L L-glucose or 4.5 g/L D-glucose, 20% FBS, supplemented L-glutamine and 10 ng/μL macrophage colony-stimulating factor (M-CSF) (PeproTech, Inc., Rocky Hill, NJ) and then were passed through a 70 μm filter to remove debris. Cells were maintained for 7 days in non-tissue coated plates to promote their differentiation into unactivated (M0) macrophages. Cells were washed in PBS, and re-plated in 1% FBS/DMEM. Culture of BMDMs was done in the absence of antibiotics.

This study was carried out in accordance with the recommendation in the Guide for the Care and Use of Laboratory Animals of the National Institutes of Health, and in agreement with the Division of Laboratory Animal Resources (DLAR) at the NYU School of Medicine. The protocol was approved by the NYU School of Medicine's Institutional Animal Care and Use Committee (IACUC); approved protocol number 140203.

This study does not use human subjects.

### Monocytes from STZ-treated mice

Mice (six) were injected intraperitoneally for five days with streptozotocin (STZ) (50 mg/kg, Sigma-Aldrich) to induce diabetes or with citrate buffer (six) to serve as a control [25]. Leukocyte subsets were identified from whole blood as previously described [29]. Monocytes were identified as CD45^hi^CD115^hi^ and Ly6-C^hi^ using the following antibodies: APC anti-mouse Ly-6G/Ly-6C (Gr-1), PE anti-mouse CD115, PE/Cy7 Anti-mouse CD45 (Biolegends).

### Quantitative real time polymerase chain reaction (qRT-PCR)

Total RNA was extracted with the RNeasy Mini Kit (Qiagen) with an additional on-column DNase digestion step. cDNA was synthesized from 1 μg of RNA using the First-Strand cDNA Synthesis Kit for Real-Time PCR (USB) and random primer mix following the manufacturer's instructions. cDNA was amplified with the SYBR Green Taq Ready Mix (USB) using MyiQ Single-Color Real-Time PCR Detection System (Bio-Rad). Cyclophilin A was used as a normalization control.

### Primers

ABCA1: (qRT-PCR)

F: 5’-GGA CAT GCA CAA GGT CCT GA-3′

R: 5’-CAG AAA ATC CTG GAG CTT CAA A-3′

ABCG1: (qRT-PCR)

Forward: 5’-CCC TCA AAG CCG TAT CTG AC-3′

Reverse: 5’-TTG ACA CCA TCC CAG CCT AC-3′

Cyclophilin A: (qRT-PCR)

Forward: 5’-GGC CGA TGA CGA GCC C-3′

Reverse: 5’-TGT CTT TGG AAC TTT GTC TGC AA-3′

Nascent ABCA1: (qRT-PCR)

Forward: 5’-TAGGATGAACCAACCACAGG-3′

Reverse: 5’-GGGCACAATTCCACAAGAAT-3′

PRMT2: (qRT-PCR)

Forward: 5’-AAGGTGCTCTTCTGGGACAA-3′

Reverse: 5’-ATGATTCGACTTTGGCCTTG-3′

ABCA1 LXRE (ChIP):

Forward: 5’-GGG GAA AGA GGG AGA GAA CAG-3′

Reverse: 5’-GAA TTA CTG GTT TTT GCC GC-3′

### Preparation of cell extracts and Western blotting

Macrophages were washed twice in ice-cold PBS prior to lysis (lysis buffer: 50 mM HEPES pH 7.6, 150 mM NaCl, 1 mM EDTA pH 8.0, 1 mM EGTA pH 8.0, 1 mM NaF, 1% Triton X-100, 10% glycerol) with protease inhibitor cocktail (1:100, Cell Signaling). Cellular debris was pelleted by centrifugation at 16,000 x g for 15 minutes at 4 ^o^C. Supernatant was collected and protein concentrations were determined by the Bradford assay (Biorad). Samples were reduced using Laemmli SDS loading buffer with beta-mercaptoethanol by incubation for 30 minutes at room temperature.

Proteins were separated on 7.5% or 4–20% gradient polyacrylamide gels, and transferred to PVDF membranes. Membranes were blocked with 5% BSA in TBS for 1 hour and incubated in primary antibody (diluted in 5% BSA/TBS) at 4°C overnight. Antibodies used were anti-ABCA1 (1:1,000, Novus 400–105), anti-HSP90 (1:500, BD 610419), anti-LXRα (1:1,000, Abcam ab41902), anti-Myc (1:2,000, Cell Signaling 2276), and anti-dimethyl-arginine, asymmetric (ASYM25) (1:1,000, EMD Millipore 07–414), and anti-BRG1 (1:1,000, Abcam ab4081). Membranes were incubated with horseradish peroxidase-conjugated secondary antibody in TBS-T at room temperature for 1 hour. The specific band was detected with a chemiluminescence assay (ECL detection reagents, Pierce) and recorded on X-ray film. 

### Mouse epigenetic chromatin modification enzymes PCR array

RNA (0.5 μg) from RAW WT macrophages cultured under high or normal glucose was reverse transcribed and 84 genes encoding enzymes known or predicted to modify DNA and histones were analyzed using the Mouse Epigenetic Chromatin Modification Enzymes RT2 Profiler PCR Array (Sabiosciences/Qiagen). The list of the genes analyzed is available at http://www.sabiosciences.com/rt_pcr_product/HTML/PAMM-085A.html. Data analysis was performed using the manufacturer’s web-based software package.

### CpG methylation assay

The EpiTect Methyl II PCR Assay (Sabiosciences/Qiagen) was used to evaluate the CpG methylation status of the *Abca1* promoter region. Primers to the CpG island in the Abca1 promoter (located at Chr4: 53172657–53173060) were purchased from Sabiosciences (EPMM107692-1A). NIH 3T3 Mouse Genomic DNA (New England Biolabs N4004S) and CpG Methylated NIH 3T3 Mouse Genomic DNA (N4005S) were used as controls. Genomic DNA was extracted using the DNEasy kit (Qiagen). Genomic DNA was subjected to mock (no enzyme), methylation-sensitive (MSRE), methylation-dependent (MDRE), and double (MSRE and MDRE) restriction enzyme digestion. After digestion, the remaining DNA was quantified by qPCR with primers that amplify the *Abca1* promoter CpG Island and the percentage unmethylated and methylated DNA was determined relative to input.

### Prmt2^-/-^ mice

Sperm from *Prmt2* ^*-/-*^ males on the C57BL/6J (B6) background were provided by Y. Herault with permission from E. Nabel [30]. Mice were re-derived using *in vitro* fertilization of WT C57BL/6 females. Heterozygote male and female offspring were used to generate *Prmt2* ^*-/-*^ mice and wild-type littermate controls. A total of 10 *Prmt2* ^*-/-*^ and 10 control littermate male mice were used in this study. The *Prmt2* ^*tm1Enbl*^ allele carries a G119X mutation and a neomycin cassette replacing exons 4 and 5 and part of exon 6 [30]. Genotyping was performed by PCR using primers: A: 5′-CTGAGGTATTACCAGCAGACA-3′; B: 5′-CTCTCTGATGCAGGTCTAC-3′; C: 5′-CCGGTGGATGTGGAATGTGT-3′. Primers A and B identify the wild-type allele (190-bp) and primers B and C identify the mutant allele (280-bp fragment).

### Cholesterol efflux

BMDMs were loaded with ^3^H-cholesterol-Ac-LDL for 24 hours (0.5 μCi/ml ^3^H-cholesterol and 50 μg/ml Ac-LDL, [1,2-^3^H(N)]-Cholesterol, NET139250UC PerkinElmer), and equilibrated in 2 mg/mL BSA media overnight. Efflux to APOAI [50 μg/ml, human APOAI; Biomedical Technologies (BT-927)], or no acceptor (all using 2 mg/ml BSA media) was done for 24 hours.

### Chromatin Immunoprecipitation (ChIP) assays

BMDMs were analyzed by ChIP as previously described [31]. Protein–DNA complexes were cross-linked in 1% formaldehyde for 10 minutes at room temperature, followed by incubation in 0.125 M glycine for 5 minutes to quench the cross-linking reaction. Cells were washed with 1x PBS twice and lysed in 5 mM PIPES at pH 8.0, 85 mM KCl, 0.5% NP-40 with protease inhibitor (Cell Signaling). The cell lysate was centrifuged at 250 x g for 5 minutes at 4°C, and the crude nuclear pellets were collected. Chromatin was sonicated into 500 bp—2,000 bp fragments using the Bioruptor (Diagenode; Twin UCD-400). Immunoprecipitation was performed overnight at 4°C using anti-LXRα (6 μg, Abcam ab41902) mouse monoclonal antibody or an equivalent amount of mouse IgG (Sigma-Aldrich). Protein G magnetic beads (Invitrogen) were used to recover antibody complexes. The beads were washed in 100 mM Tris at pH 7.5, 500 mM LiCl, 1% NP-40, 1% sodium deoxycholate and TE (10 mM Tris-HCl at pH 7.5, 0.1 mM Na_2_EDTA) at 4°C. Following reversal of cross-linking, recovered DNA was purified using PrepEase DNA Clean-Up Kit (USB). Cycle threshold values were normalized to percent input and IgG.

### Statistical analysis

Unless otherwise noted, all quantitative results are averages from at least three independent experiments, and each sample was analyzed in triplicate. Data are expressed as the mean ± SEM. Significance was determined using the two-tailed Student's t-test (*, P < 0.05; **, P < 0.01; ***, P < 0.001). All tests were performed using the Prism software (GraphPad Software, Inc., La Jolla, CA). Western blots are representative of at least three independent experiments.

## Results

### High glucose inhibits LXR-mediated induction of Abca1 and decreases ABCA1-dependent cholesterol efflux

**Fig 1:**
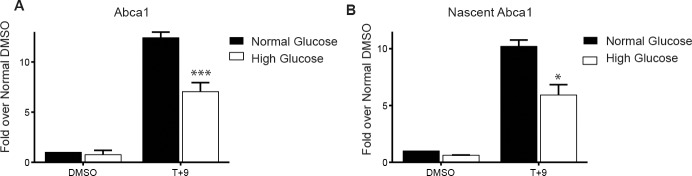
High glucose inhibits induction of *Abca1* in RAW macrophages. RAW WT macrophages were treated for four hours with 5 μM T + 1 μM 9cisRA (T+9) or DMSO vehicle control and steady state RNA (A) and nascent RNA (B) transcripts of *Abca1* were profiled using qRT-PCR. *Cyclophilin A* was used for normalization. Macrophages were cultured overnight in 1% FBS prior to treatment. Panel A represents an average of five independent experiments and panel B represents an average of three independent experiments. Error bars indicate the SEM. Significance is determined using the two-tailed Student's t-test (*, P < 0.05, **, P<0.01, ***, P<0.001).

To investigate the effect of glucose levels on LXR-mediated gene expression in macrophages, RAW cells stably expressing human LXRα (RAW WT) were maintained in diabetes relevant high glucose (25 mM D-glucose; 4.5 g/L D-glucose) or normal glucose (5.5 mM D-glucose; 1 g/L D-glucose supplemented with 3.5 g/L of L-glucose as an osmotic control) for two weeks. Cells were treated with LXR/RXR ligands [TO901317 + 9cis retinoic acid: (T+9)] or DMSO as a vehicle control for four hours and expression of the canonical LXR target gene *Abca1* was determined. Ligand treatment robustly induced *Abca1* expression in RAW WT under normal glucose conditions. This induction was significantly reduced in RAW WT cells cultured under high glucose conditions (Fig 1A). Many processes, both transcriptional and post-transcriptional, regulate steady state mRNA levels. To identify if the decrease in *Abca1* mRNA was transcriptional, we also measured newly formed heteronuclear, or nascent, RNA. The change observed in steady state mRNA was reflected in the nascent RNA transcripts of ABCA1: there was less nascent *Abca1* mRNA production in high compared to normal glucose (Fig 1B). This suggests that the differences in mRNA as a function of high glucose are due to a defect in LXR-mediated transcription of the *Abca1* gene.

**Fig 2:**
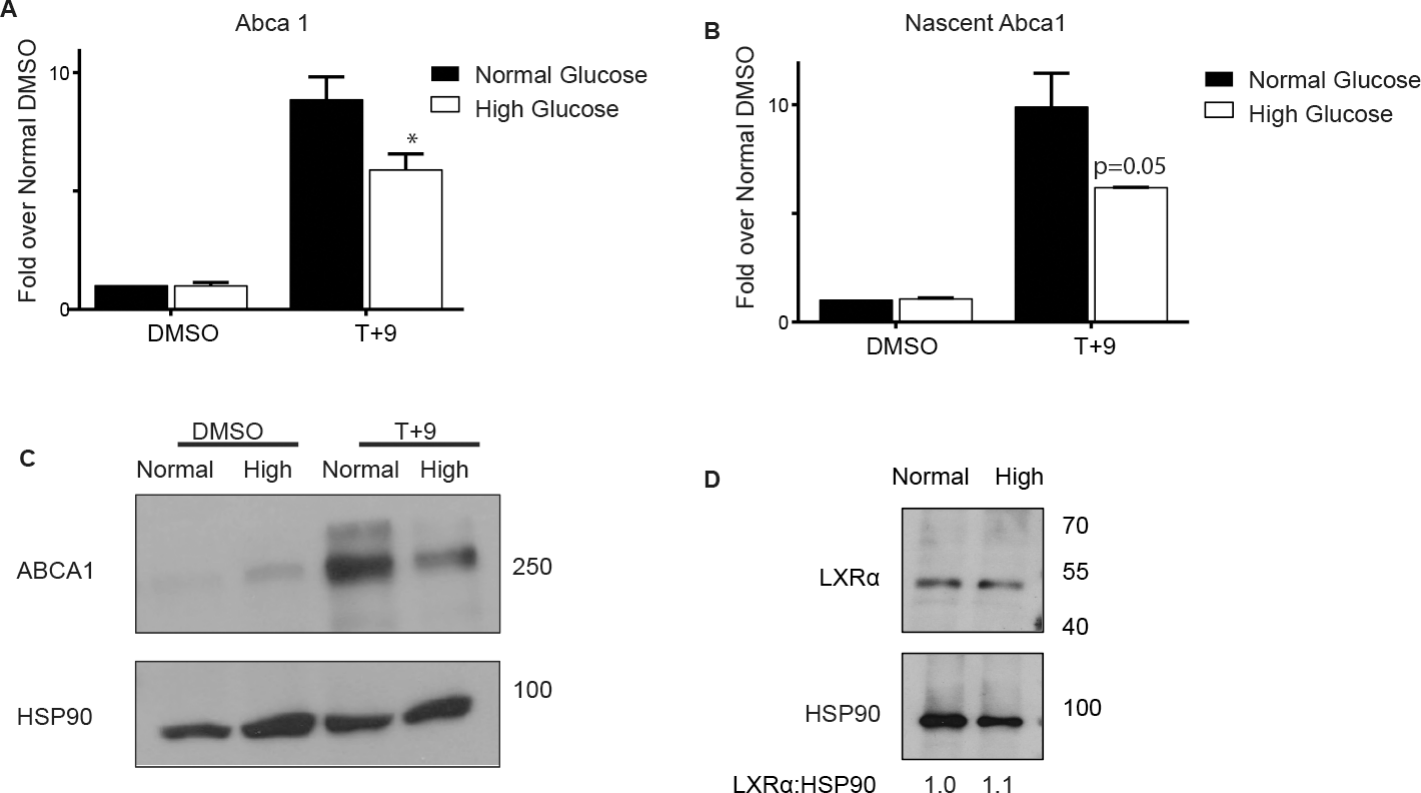
High glucose inhibits induction of *Abca1* in BMDMs. Bone marrow cells from C57BL/6 mice were differentiated into macrophages under high (25 mM D-glucose) or normal (5.5 mM D-glucose + 19.5 mM L-glucose) glucose. Prior to treatments, macrophages were cultured in 1% FBS overnight and then treated for four hours with 5 μM T + 1 μM 9cisRA (T+9) or DMSO vehicle control and steady state RNA (A) and nascent RNA (B) transcripts of *Abca1* were profiled using qRT-PCR. *Cyclophilin A* was used for normalization. (C) BMDMs were cultured and treated as in A for eight hours. Whole cell protein extracts were collected and separated on a 7.5% polyacrylamide gel and were blotted for ABCA1. HSP90 was used as a loading control. (D) Whole cell protein extracts collected from untreated BMDMs cultured under high and normal glucose were separated as in (C) and blotted for LXRα. HSP90 was used as a loading control. The levels of LXRα and HSP90 protein were quantitated by densitometry and presented below with normal glucose set to 1. The ratio of panels A and B represent an average of three independent experiments. Error bars indicate the SEM. Significance is determined using the two-tailed Student's t-test (*, P < 0.05, **, P<0.01, ***, P<0.001). (C) and (D) are representative.

To ensure that this is not a model-specific effect, we repeated the experiment using bone marrow derived macrophages (BMDMs) differentiated in high or normal glucose, and treated with LXR/RXR ligands. As in RAW WT cells, high glucose impaired ligand-mediated up-regulation of *Abca1* in BMDMs (Fig 2A), and this defect was reflected in the production of nascent mRNA transcripts (Fig 2B). Importantly, the changes in mRNA translated to a decrease in ABCA1 protein (Fig 2C). In contrast, the levels of LXRα protein were similar under both glucose conditions (Fig 2D), which suggest that the changes in *Abca1* expression are due to a defect in LXR function as opposed to an effect of glucose on the expression of LXR itself. Interestingly, the expression of *Abcg1*, another canonical LXR target gene, was not influenced by changes in glucose levels (S1 Fig). This suggests an effect of glucose on an LXR cofactor that confers a gene specific effect.

**Fig 3:**
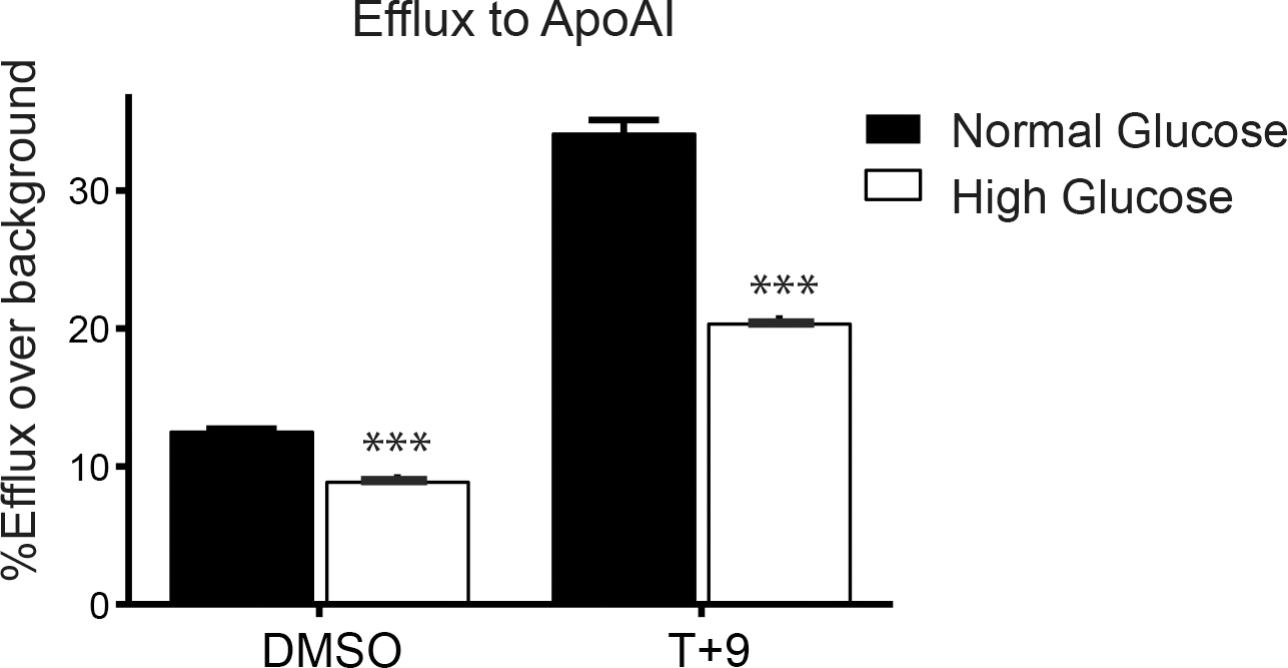
Cholesterol efflux to APOAI is inhibited by high glucose. Bone marrow cells from C57BL/6 mice were differentiated into macrophages under high (25 mM D-glucose) or normal (5.5 mM D-glucose + 19.5 mM L-glucose) glucose. BMDMs were loaded with ^3^H-cholesterol-Ac-LDL for 24 hours (0.5 μCi/mL ^3^H-cholesterol and 50 μg/mL Ac-LDL), and then equilibrated in 2 mg/mL BSA media overnight. During equilibration cells were treated with 5 μM T + 1 μM 9cisRA or DMSO vehicle control. Efflux to APOAI (50 μg/mL), or no acceptor (all using 2 mg/mL BSA media) was done for eight hours. Specific efflux to APOAI is represented as a percentage of total efflux over background efflux to no acceptor. Experiment was performed in quadruplicate. Error bars represent SEM. Significance is determined using the two-tailed Student's t-test (*, P < 0.05, **, P<0.01, ***, P<0.001).

To assess the functional effect of high glucose-driven reduction in ABCA1 protein, we utilized a cholesterol efflux assay. BMDMs cultured under normal or elevated glucose conditions were loaded with ^3^H-cholesterol-Ac-LDL for 24 hours and their ability to efflux cholesterol was assayed by the addition of the cholesterol acceptor APOAI to the media (Fig 3). Under normal glucose conditions, robust APOAI-associated cholesterol efflux was observed when compared to a control (no acceptor) condition that served to measure background efflux. Notably, under high glucose conditions there was a significant reduction in ABCA1-mediated efflux to APOAI (from 12.5% in normal glucose to 8.9% in high glucose, a 29.0% reduction). This significant decrease in APOAI-directed efflux under high glucose was also seen in the presence of the synthetic LXR/RXR ligands (T+9), which are known to further enhance ABCA1 expression above that of Ac-LDL alone (normal glucose, 34.1%; high glucose, 20.3%; a 40.0% reduction) [32]. Thus, the reduction of ABCA1 gene expression under high glucose conditions resulted in a functional decrease in ABCA1-dependent cholesterol efflux.

### A subset of chromatin modifying enzymes is differentially expressed under high glucose conditions

**Fig 4:**
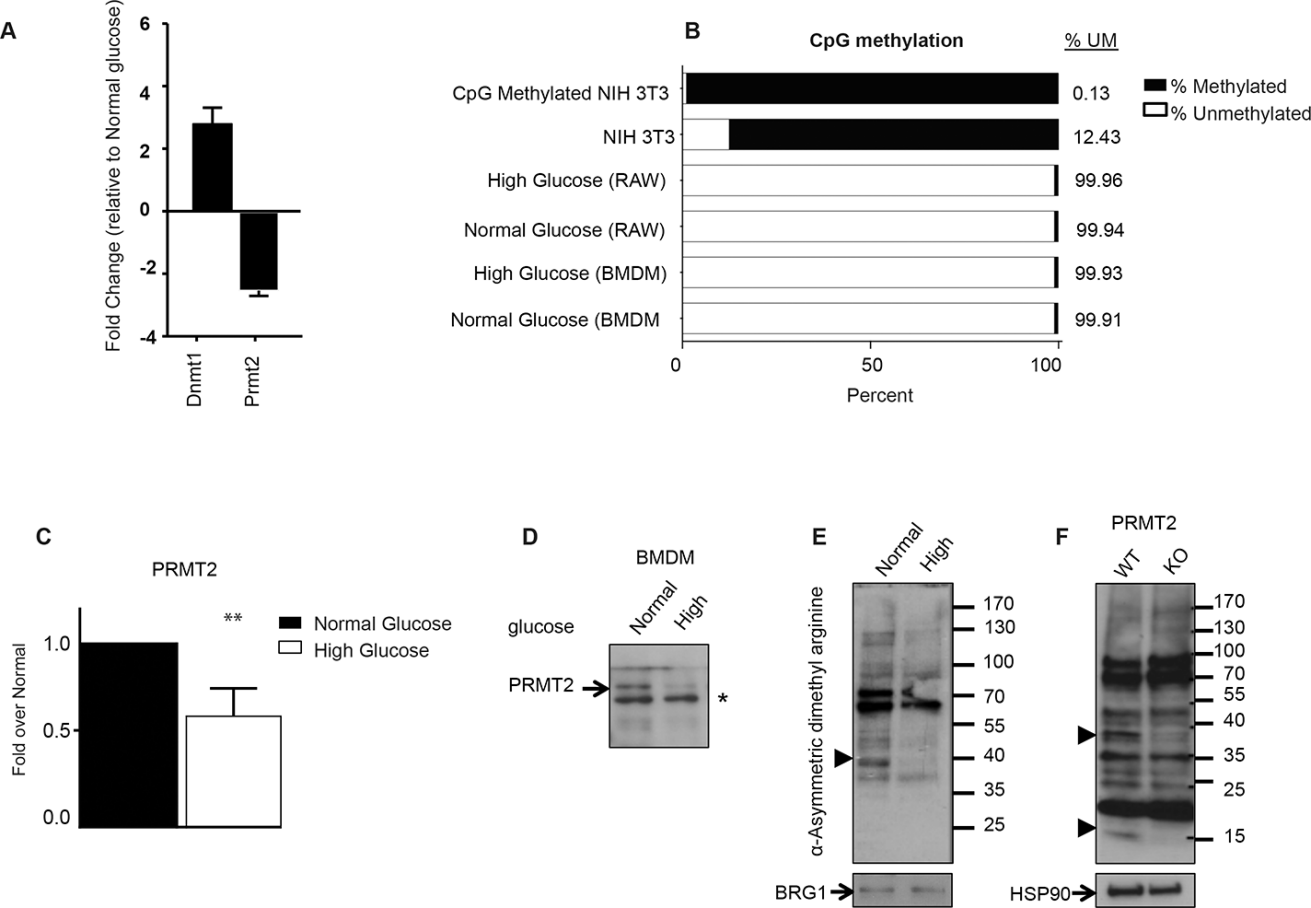
Chromatin-modifying enzymes expression and *Abca1* CpG island methylation under high and normal glucose in macrophages. (A) RNA from RAW WT cells maintained under high (25 mM D-glucose) or normal (5.5 mM D-glucose + 19.5 mM L-glucose) glucose was profiled using a qRT-PCR-based chromatin-modifying array and expression changes in DNMT1 and PRMT2 are shown. (B) Epitect methyl CpG assay was employed to examine the methylation status of the *Abca1* promoter CpG island. NIH 3T3 Mouse Genomic DNA and CpG Methylated NIH 3T3 Mouse Genomic DNA were used as controls. Input DNA was cleaved with methylation-sensitive and/or methylation-dependent restriction enzymes that digest unmethylated and methylated DNA, respectively. Following digestion, remaining DNA was quantified by qRT-PCR using primers that flank the CpG island. Percent methylation was determined by comparing the amount digested by each enzyme to a mock digested sample. (C) RNA from BMDMs differentiated under high (25 mM D-glucose) or normal (5.5 mM D-glucose + 19.5 mM L-glucose) glucose was extracted and levels of *Prmt2* were profiled using qRT-PCR. (D) PRMT2 protein expression in normal and high glucose. Nuclear extract of BMDMs cultured as in C were blotted for PRMT2. Asterisk (*) represents a non-specific band, which serves as a loading control. (E) Nuclear protein extracts from BMDMs from wild type (WT) mice cultured in normal or high glucose and (F) whole cell extracts from BMDMs from wild type (WT) and *Prmt2* ^-/-^ (KO) mice cultured under normal glucose were separated on a 4–20% gradient polyacrylamide gel and immunoblotted with an antibody specific for asymmetric dimethyl arginine. BRG1 or HSP90, serve as nuclear and whole-cell loading controls, respectively. Filled arrowheads represent asymmetric dimethyl arginine immunoreactive proteins uniquely present in one of the conditions.

Our data suggest that the mechanism of varying ABCA1 expression as a function of glucose levels is at the level of transcription. Previous studies have shown that hyperglycemia induces genome wide changes in histone modifications and DNA methylation in aortic endothelial cells [33, 34]. We examined whether glucose concentration affected the expression of enzymes that are known to regulate transcription by altering the modifications on DNA and histones in macrophages. We cultured RAW WT macrophages under high and normal glucose conditions, extracted RNA and performed a qRT-PCR based array that examines the expression of 84 genes encoding enzymes that modify DNA and histones to regulate chromatin architecture. A subset of enzymes showed changes in gene expression >2 fold under the different glucose conditions. These included DNMT1, a maintenance DNA methylation enzyme that displayed increased expression in high glucose, and PRMT2, a type I protein arginine methyltransferase that showed decreased expression in high glucose (Fig 4A). Type I enzymes asymmetrically dimethylate a single terminal nitrogen residue of proteins [35]. Changes in CpG methylation in aortic endothelial cells cultured under high glucose and recognition of PRMT2 as a transcriptional coactivator for other nuclear receptors made these attractive candidates to modulate ABCA1 expression as a function of glucose [26, 27, 33].

The level of DNMT1 is higher in RAW WT cells cultured under high glucose, potentially conferring a repressive effect on transcription. There is also a CpG island in the *Abca1* promoter that is conserved between humans and mice [36]. Given DNMT1’s role in maintaining CpG methylation throughout cell divisions and precedence for glucose leading to differences in promoter methylation we tested if DNA methylation of the *Abca1* promoter is modulated by glucose. We probed the methylation status of the CpG island of *Abca1* in both RAW WT cells and BMDMs cultured under high and normal glucose using methylation-dependent and-sensitive restriction enzyme digests coupled with qPCR detection. To establish the efficacy of the assay at the *Abca1* CpG island we first examined genomic DNA from NIH 3T3 cells (a mouse embryonic fibroblast cell line) that was enzymatically fully methylated with CpG methylase and compared that to genomic DNA from control NIH 3T3 cells that should naturally have a mixture of methylated and unmethyalted DNA. We observed from control NIH 3T3 cell DNA that 12.40% of the *Abca1* CpG island was unmethylated, while this number dropped to 0.13% in the *in vitro* fully methylated DNA (Fig 4B). Thus the assay can effectively distinguish between alterations in the DNA methylation status of the *Abca1* promoter. Applying this assay to genomic DNA from RAW WT cells and BMDMs cultured under high and normal glucose conditions, we determined that RAW WT cells under high glucose had 99.96% unmethylated CpGs while CpGs in RAW WT cells under normal glucose were 99.94% unmethylated. Similarly BMDMs under high glucose were 99.93% unmethylated and BMDMs under normal glucose were 99.91% unmethylated (Fig 4B). The *Abca1* promoter was nearly fully unmethylated in both cell types. This provides evidence that the CpG methylation status of *Abca1* does not govern the differences in *Abca1* expression upon changes in glucose concentrations, and further does not implicate a major role for DNMT1 in this process.

### Modulation of PRMT2 levels affects Abca1 expression

We were interested in the role of PRMT2 as a potential LXR regulator as it has been shown to bind RXRα, the obligate binding partner of LXR [27]. Further, it has been shown to enhance the transcriptional activity of other nuclear receptors, including AR and ERα [26, 27]. Significantly, the decrease in *Prmt2* expression under high glucose seen in RAW WT cells was also observed in BMDMs at the mRNA (Fig 4C) and protein level (Fig 4D), which strengthens the potential regulatory role of this enzyme in LXR function.

By homology PRMT2 has been classified as a Type I enzyme responsible for asymmetrically dimethylating target proteins [35, 37]. However the *in vivo* methyltransferase activity of PRMT2 has been thus far difficult to demonstrate [38]. Therefore, we examined globally for the effect of PRMT2 on protein arginine methylation using an antibody that recognizes asymmetric dimethylarginine. We examined protein extracts from BMDMs cultured in high vs. normal glucose as well as from *Prmt2* ^*-/-*^ macrophages and blotted for asymmetric dimethylarginine. There were changes in methylated protein substrates from nuclear extracts in wild type BMDMs cultured in high vs. normal glucose (Fig 4E) as well as specific changes in the methylome from whole cell extracts between wild type and *Prmt2* ^*-/-*^ macrophages (Fig 4F). This suggests that PRMT2 impacts protein arginine methylation and that a subset of proteins appear differentially methylated as a function of glucose concentrations.

**Fig 5:**
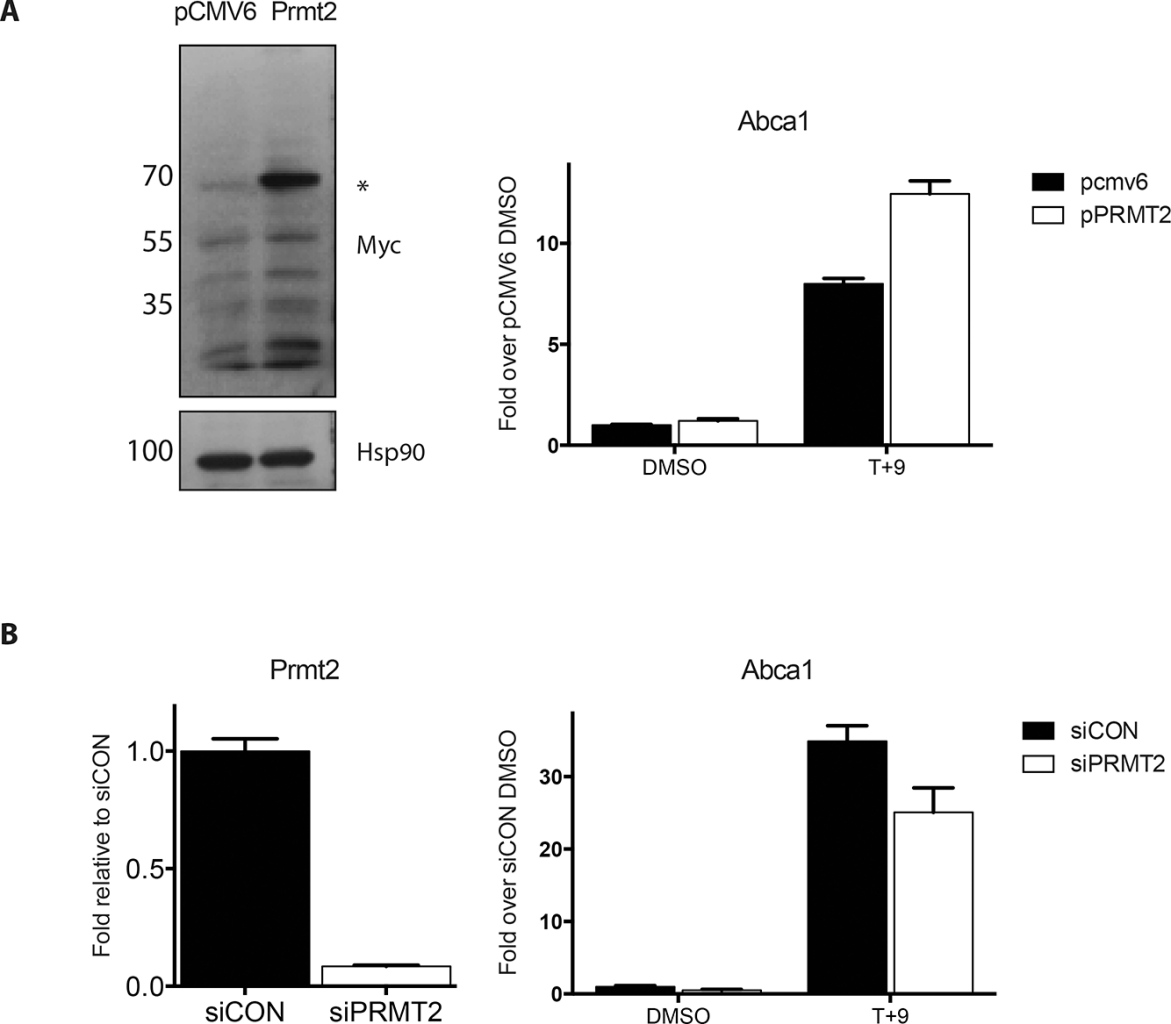
*Prmt2* levels affect ligand-induced expression of *Abca1*. (A) Myc-DDK tagged PRMT2 in the pCMV6 expression vector, or an empty pCMV6 (vector) control were transfected into RAW WT macrophages. Overexpression was confirmed by blotting for the Myc-tag on PRMT2 or HSP90 as a loading control. One day following transfection macrophages were cultured in 1% FBS overnight and then treated for four hours with 5 μM T +1 μM 9cisRA (T+9) or DMSO vehicle control. RNA was extracted and *Abca1* mRNA was measured by qRT-PCR. (B) PRMT2 levels were depleted in RAW WT cells using siRNA specific to *Prmt2* (siPRMT2) or scrambled siRNA as a control (siCON). *Prmt2* depletion at the mRNA level was confirmed by qRT-PCR. Following knockdown, cells were treated as in (A) and *Abca1* mRNA expression was determined by qRT-PCR. *Cyclophilin A* was used as a control to normalize the qRT-PCR reactions. Experiments were performed twice. Error bars represent SD of three technical replicates.

Having established that the levels of *Prmt2* mRNA are lower in macrophages cultured in high glucose relative to normal glucose concentrations, we next investigated the effects of increased levels of PRMT2 on LXR-dependent transcriptional activity in RAW macrophages. Transient overexpression of PRMT2 increased LXR/RXR ligand-dependent transcription of *Abca1* by about 50% (Fig 5A), without affecting *Abcg1* expression (S1 Fig). By contrast, depletion of PRMT2 in RAW WT cells using siRNAs specific to *Prmt2* decreased ligand-dependent *Abca1* mRNA expression (Fig 5B), consistent with PRMT2 possessing coregulatory function for LXR at *Abca1*.

**Fig 6:**
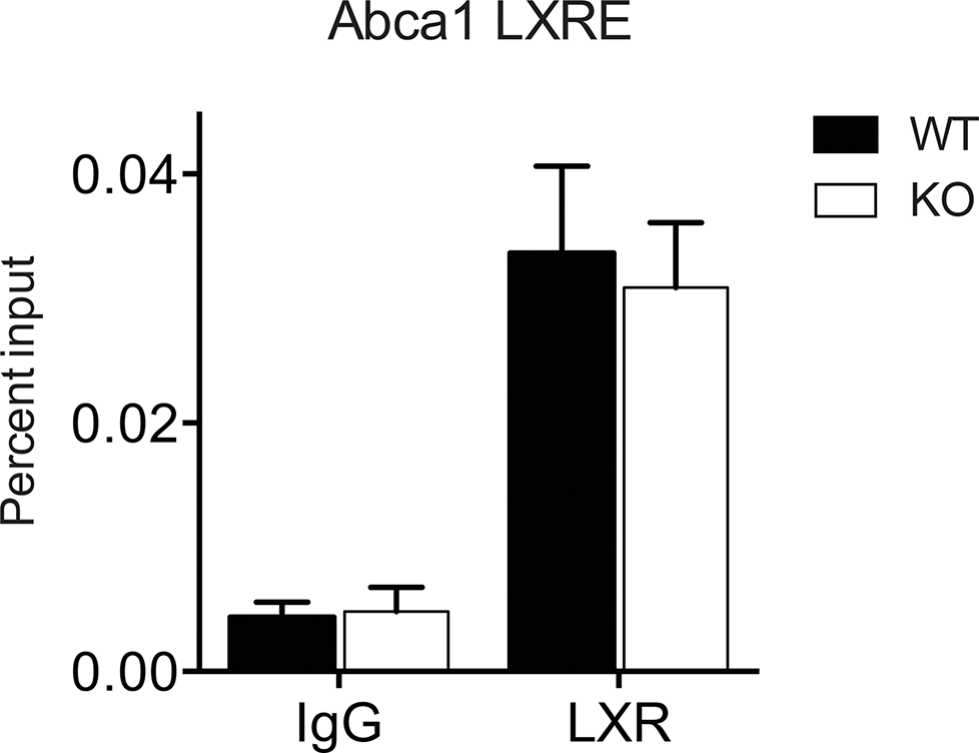
LXRα occupancy of *Abca1* promoter LXRE. Chromatin was immunoprecipitated from wild type or *Prmt2* ^*-/-*^ BMDMs treated for one hour with T+9 using an antibody specific to LXRα. Isotype matched IgG was used as a control. Percent of precipitated DNA compared to total input DNA is shown. Data represent an average of three independent experiments. Error bars represent SEM. Difference in LXRα occupancy between WT and *Prmt2* ^*-/-*^ was not significant.

PRMTs have been shown to have histone-modifying functions, which affect transcription factor binding [39]. Given this, we examined whether LXRα occupancy at the *Abca1* promoter was affected by *Prmt2* deletion by chromatin immunoprecipitation (ChIP) of LXRα from ligand-treated wild type vs. *Prmt2* ^*-/-*^ BMDMs. We did not observe any significant differences in LXRα occupancy in *Prmt2* ^*-/-*^ compared to wild type cells (Fig 6). Similar results were observed using an antibody that recognizes both LXRα and LXRβ (data not shown). This indicates that the mechanism of the PRMT2 effect on LXR-mediated transcription of *Abca1* is not a result of differences in LXR occupancy at its binding site in the *Abca1* promoter.

### PRMT2 knockout macrophages show defects in upregulation of ABCA1 and decreased cholesterol efflux to APOAI

**Fig 7:**
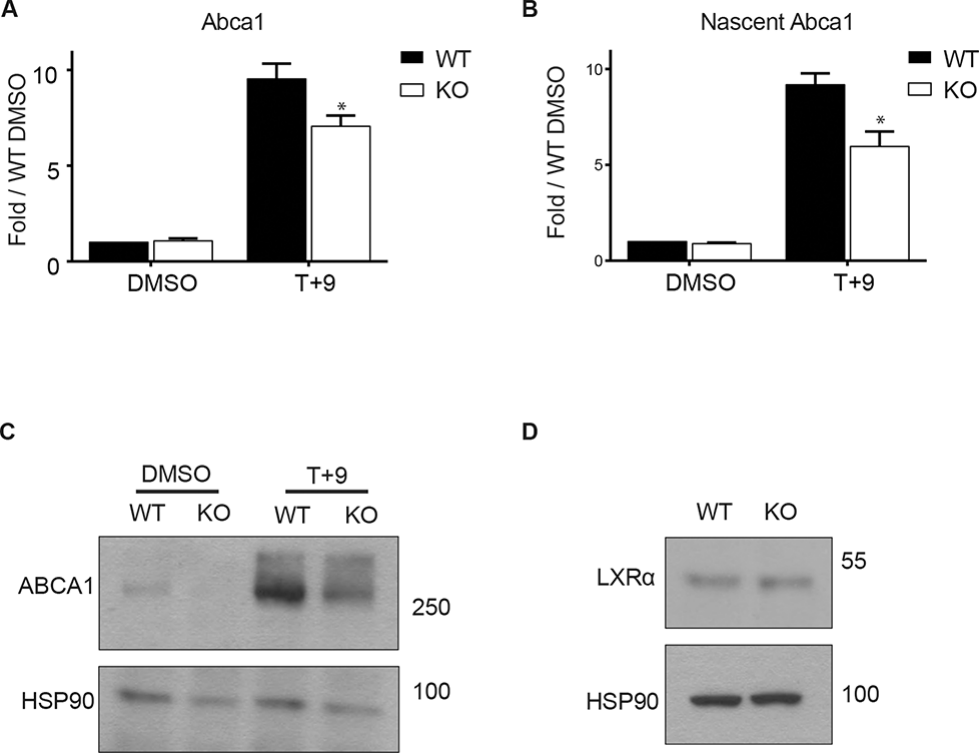
*Prmt2* ^*-/-*^ BMDMs mimic the effect of high glucose on ABCA1. BMDMs from wild type (WT) or *Prmt2* ^*-/-*^ (KO) mice were differentiated under normal glucose (5.5 mM D-glucose) conditions. Following differentiation BMDMs were cultured in 1% FBS overnight and then treated for four hours with 5 μM T + 1 μM 9cisRA (T+9) or DMSO vehicle control. RNA was collected and steady state (A) or nascent (B) *Abca1* levels were determined by qRT-PCR. *Cyclophilin A* was used as a control. (C) Whole-cell protein extracts from BMDMs cultured as in A and treated for eight hours with T+9 or DMSO were separated on a 7.5% polyacrylamide gel and immunoblotted for ABCA1 or HSP90 (loading control). (D) Whole-cell protein extracts from WT and KO BMDMs cultured under normal (5.5 mM D-glucose) glucose were separated on 7.5% polyacrylamide gel and immunoblotted for LXRα. HSP90 was used as a loading control. (A) and (B) represent averages of three independent experiments. Error bars indicate the SEM. Significance is determined using the two-tailed Student's t-test (*, P < 0.05, **, P<0.01, ***, P<0.001).

To further explore the role of PRMT2 in macrophage *Abca1* expression and cholesterol efflux, we carried out studies using BMDMs from *Prmt2* deficient mice [30]. *Prmt2* ^*-/-*^ mice lack any morphological or observable developmental defects [30]. As in previous experiments, BMDMs were treated for four hours with LXR/RXR ligands (T+9). Compared to macrophages from wild type littermate control mice, there was a pronounced reduction in *Abca1* mRNA induction (steady state and nascent) upon ligand stimulation (Fig 7A and 7B). This difference was also evident at the protein level wherein *Prmt2* ^*-/-*^ macrophages had less ABCA1 than wild type under ligand-stimulated conditions (Fig 7C). Importantly, LXR protein levels were equal in wild type and *Prmt2* ^*-/-*^ macrophages, as was also seen in BMDMs cultured under high or normal glucose (Fig 7D and 7D). *Abcg1* expression was not affected by depletion of PRMT2, which is consistent with the observed lack of a glucose effect on *Abcg1* expression (S1 Fig). We also examined expression of the LXR target genes *Srebp1c*, *Lpl*, *Apoe* from WT and *Prmt2* ^*-/-*^ BMDMs treated with T+9 and found that they did not demonstrate a change in expression as a function of PRMT2 (S2 Fig). This suggests that PRMT2 imparts gene-specific effects on LXR target gene expression upon T+9 treatment.

**Fig 8:**
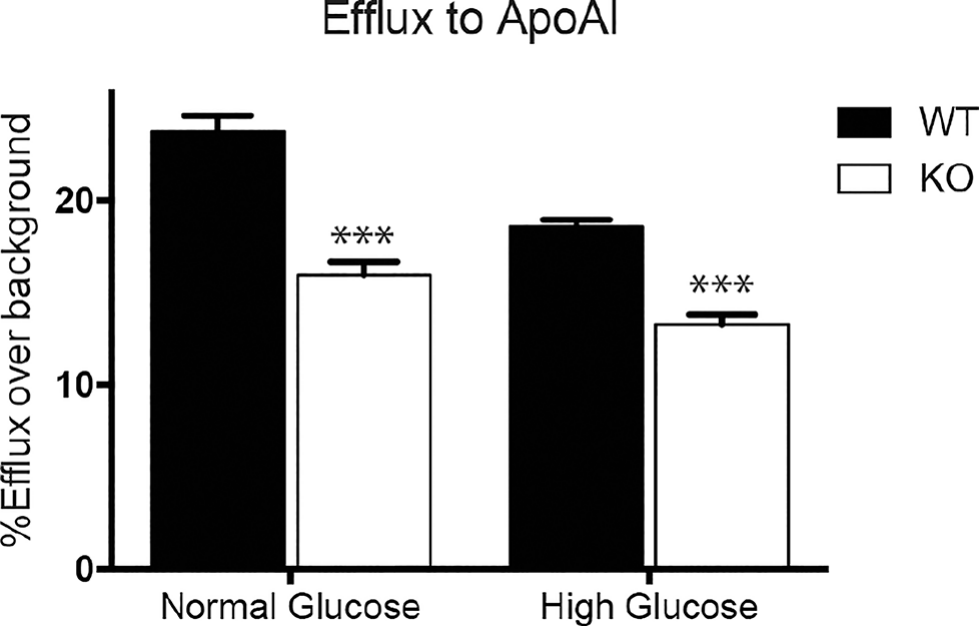
Efflux to APOAI is reduced in *Prmt2* ^*-/-*^ BMDMs. Wild type and *Prmt2* ^*-/-*^ BMDMs were differentiated under high glucose (25 mM D-glucose) or normal glucose (5.5 mM D-glucose + 19.5 mM L-glucose) and following differentiation were loaded with ^3^H-cholesterol-Ac-LDL for 24 hours (0.5 μCi/mL ^3^H-cholesterol and 50 μg/mL Ac-LDL), and then equilibrated in 2 mg/mL BSA media overnight. Efflux to APOAI (50 μg/mL), or no acceptor (all using 2 mg/mL BSA media) was done for 24 hours. Specific efflux to APOAI is represented as a percentage of total efflux over background efflux to no acceptor. Experiment was performed in quadruplicate. Error bars represent SEM. Significance is determined using the two-tailed Student's t-test (*, P < 0.05, **, P<0.01, ***, P<0.001).

We next assessed whether the reduction in ABCA1 in *Prmt2* ^*-/-*^ macrophages affected cholesterol efflux. We found that there was a marked reduction in ABCA1-mediated cholesterol efflux to APOAI in *Prmt2* ^*-/-*^ macrophages when compared to wild type littermate control macrophages (Fig 8). This reduction in efflux was observed under normal glucose (WT, 23.7% efflux; KO, 15.9% efflux) and in high glucose albeit to a lesser extent (WT, 18.6% efflux, KO, 13.2% efflux). This suggests PRMT2 is an important mediator of ABCA1 expression and cholesterol efflux in primary macrophages, and that in addition to PRMT2-dependent effects, high glucose imparts PRMT2-independent effects on cholesterol efflux.

### Prmt2 levels are decreased in monocytes from diabetic mice

**Fig 9:**
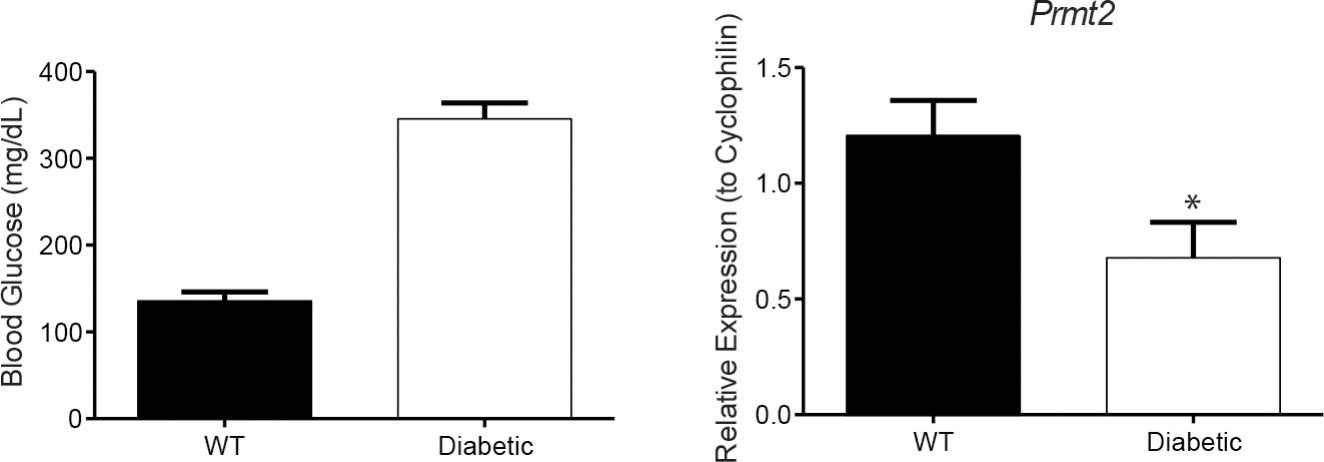
*Prmt2* expression is reduced in monocytes from diabetic mice. (A) Blood glucose levels were measured from wild type and STZ-treated mice. (B) The level of PRMT2 in Ly6-C^hi^ monocytes from wild type and STZ-treated mice was profiled using qRT-PCR. *Cyclophilin A* was used as an endogenous control. Six mice were used per condition. Error bars represent SEM. Significance is determined using the two-tailed Student's t-test (*, P < 0.05).

To determine if high glucose affects the expression of *Prmt2 in vivo*, monocytes (Ly6-C^hi^) from C57BL/6, STZ-treated diabetic mice were isolated and *Prmt2* expression was determined. As expected, mice treated with STZ showed elevated blood glucose levels when compared to control-treated mice (Fig 9A). Importantly, compared to the non-diabetic control mice, diabetic mice showed decreased expression of *Prmt2* in Ly6-C^hi^ monocytes (Fig 9B), the subset that constitutes the largest fraction of monocytes recruited to mouse atherosclerotic plaques [40]. This suggests that hyperglycemia affects *Prmt2* expression *in vivo*.

## Discussion

This study explores the mechanism whereby diabetes-relevant high glucose modulates LXR-dependent gene expression in macrophages as a prelude to understanding the clinical observation that diabetes exacerbates atherosclerosis. We demonstrate that in macrophage-like cell lines and primary bone marrow derived macrophages cultured under high glucose there is a selective reduction in the LXR-dependent induction of ABCA1 compared to cells cultured under normal glucose concentrations upon activation by the combination of LXR/RXR ligands T+9. This results in a functional effect of reduced ABCA1-dependent cholesterol efflux to APOAI under high vs. normal glucose. We identified PRMT2 as a glucose-sensitive factor that impacts LXR’s ability to regulate expression of ABCA1. PRMT2 mRNA expression and protein levels are reduced when macrophages are cultured in high as compared to normal glucose. Upon overexpression PRMT2 enhances *Abca1* expression, whereas its depletion reduces *Abca1* gene expression, with T+9 treatment. Interestingly we also found that *Prmt2* ^-/-^ macrophages express less *Abca1* and are less effective at ABCA1-mediated efflux to APOAI compared to wild type macrophages with T+9 stimulation. Interestingly, the expression of *Abca1* with individual LXR/RXR ligands was less sensitive to changes in PRMT2 expression (S3 Fig), suggesting that the action of PRMT2 is most relevant when both LXR and RXR are activated. These findings support a role for PRMT2 in LXR-mediated regulation of ABCA1 expression and cholesterol efflux as a function of glucose concentrations and ligands. Furthermore, this suggests a potential molecular explanation behind enhanced atherosclerosis in diabetic patients. It will be interesting to test additional LXR ligands for effects on the expression of *Abca1* and other LXR target genes as a function of PRMT2 expression in future studies.

Studies by Maio *et al*. identified genes differentially expressed in normal vs. high glucose in human THP-1 cells by RNA-seq [41], but did not report PRMT2 as a gene that was down regulated in high glucose. This could reflect differences in experimental design (acute high glucose stimulation in their study vs. chronic high glucose treatment in ours) or cell type (undifferentiated THP-1 monocytes vs. differentiated BMDMs). Studies in differentiated THP-1 cells exposed to chronic high glucose concentrations could be done to examine this apparent discrepancy.

PRMTs have been shown to modulate nuclear receptor activity through a number of mechanisms in addition to their histone-modifying functions. For example, the hepatocyte nuclear factor (HNF4) is a nuclear receptor that is methylated by PRMT1, and methylation of the HNF4 DNA-binding domain enhances HNF4’s affinity for its cognate binding site [42]. The mechanism by which PRMT2 exerts its role on *Abca1* upregulation by LXR is unknown. PRMT2’s effect on *Abca1* occurs only in the presence of ligand, suggesting that the presence of increased PRMT2 alone is insufficient to increase *Abca1* expression levels. This was also true in PRMT2-mediated coactivation of ERα [27]. Regulation of LXR-dependent expression of *Abca1* could be mediated via PRMT2’s ability to modify histone or non-histone proteins to affect transcriptional activity. However, we did not observe alterations in the methylation of histone H3 arginine 8 (H3R8), the putative target of PRMT2, at the *Abca1* promoter in wild type or *Prmt2* ^-/-^ macrophages, nor did we observe arginine methylation of LXRα as a function of PRMT2 expression (data not shown). Interestingly, the impact of both glucose and PRMT2 is LXR gene specific: for example, the LXR target gene *Abcg1* is not affected by depletion of PRMT2 nor is *Abcg1* expression influenced by changes in glucose levels (S1 Fig).

*Abca1* expression is controlled not only by LXRs but also by cAMP and protein kinase A-dependent activation of CREB1. Elegant work from the Smith laboratory defined a cAMP responsive element within the first intron of the mouse *Abca1* locus that is bound by activated CREB1 [43]. However, the finding that high glucose impacts the regulation of *Abca1* via CREB1 is unlikely given that CREB1 appears to be activated by high glucose [44], and would therefore be predicted to increase rather than decrease *Abca1* expression.

Recent molecular analysis of coregulator assembly at *Abcg1* and *Abca1* uncovered distinct mechanisms of LXR regulation that may offer insight to the finding that the expression of *Abca1*, but not *Abcg*1 is affected by high glucose. Jakobsson *et al*. found that the recruitment of LXR to the *Abcg*1 promoter/enhancer unit is ligand-dependent and requires the coregulator G-protein pathway suppressor 2 (GPS2), in conjunction with histone demethylases [KDM1 (LSD1), KDM3A (JHDM2A), and KDM4A (JMJD2A/JHDM3A)], that relaxes chromatin structure to promote gene expression [45]. This is in contrast to *Abca1* where LXR is pre-bound to the *Abca1* promoter in the absence of ligand along with GPS2, NCOR and HDAC2, which serve to repress its expression. Whereas GPS2 is a coactivator at *Abcg1* and is recruited to the promoter upon ligand treatment, it is a co-repressor at *Abca1* and is dismissed upon ligand stimulation. Whether PRMT2 affects the activity of these or other coregulators in high vs. normal glucose via arginine methylation to promote co-repressor or block co-activator binding remains an open question. Indeed, factors that are differentially arginine methylated in normal compared to high glucose (Fig 4E) could represent candidates that drive differential gene expression of *Abca1* as a function of glucose.

A clear mechanism for the differential gene sensitivity of *Abca1* and *Abcg1* toward PRMT2 could reflect differences in the occupancy of PRMT2 at one gene but not the other, which could be addressed using ChIP assays. Unfortunately, we have been unable to specifically ChIP PRMT2 with multiple commercial or in-house developed antibodies as we see similar “occupancy” of PRMT2 at *Abca1* in wild type and *Prmt2* ^*-/-*^ BMDMs. Thus, the available PRMT2 antibodies do not accurately reflect occupancy of PRMT2 via ChIP. To circumvent the lack of a ChIP grade PRMT2 antibody we plan to engineer a FLAG-epitope into the endogenous PRMT2 protein using CRISPR-Cas9 technology, and then perform ChIP assays at *Abca1* and *Abcg1* in normal and high glucose.

Little is known about what regulates *Prmt2* expression. Examination of the human protein atlas reveals expression of PRMT2 in 33 of 83 normal tissues including expression in bone marrow, lymph node, tonsils and spleen [46]. Predicted transcription factor binding sites in the promoter region of *Prmt2* indicates potential STAT, NFκB and cJun binding elements, and interrogation of the ENCODE database reveals occupancy of STAT and Jun family members in the *Prmt2* promoter, but the functionality of these factors in *Prmt2* mRNA expression has not been determined [47]. Establishing how *Prmt2* is regulated and how glucose controls *Prmt2* expression will be important for developing strategies to restore PRMT2 levels in the diabetic setting.

Given our findings that macrophages show decreased ABCA1-mediated efflux upon reduction of PRMT2, studies that examine the effects of PRMT2 directly on atherosclerosis and diabetes are now warranted but beyond the scope of this study. We would predict that mice with macrophages devoid of PRMT2 would be more prone to atherosclerosis due to their inability to efficiently efflux cholesterol. Work performed by other groups suggests a somewhat different scenario where deletion of PRMT2 has a protective effect with respect to atherosclerosis as *Prmt2* ^*-/-*^ mice fed a high fat diet were resistant to weight gain and had favorable lipid profiles, glucose tolerance tests and insulin levels [48]. However, this was done in the whole-body PRMT2 knockout mouse and is likely a result of reduced food intake by changes in leptin signaling due to PRMT2 loss in the brain. Thus, future studies directed at elucidating PRMT2 substrates, regulation and *in vivo* function in macrophages would be crucial for understanding the role PRMT2 plays in LXR-mediated regulation of *Abca1* in diabetes and atherosclerosis.

## Supporting Information

S1 Fig Ligand stimulated induction of Abcg1 is unchanged by high glucose and PRMT2 levels. (A) Bone marrow cells from C57BL/6 mice were differentiated into macrophages under high glucose (25 mM D-glucose) or normal glucose (5.5 mM D-glucose + 19.5 mM L-glucose). Prior to treatments, macrophages were cultured in 1% FBS overnight and then treated for four hours with 5 μM T + 1 μM 9cisRA or DMSO vehicle control and steady state RNA transcripts of *Abcg1* were profiled using qRT-PCR. (B) BMDMs from wild type and *Prmt2* ^-/-^ mice were cultured and treated as in (A). (C) Myc-DDK tagged PRMT2 was transfected into RAW WT macrophages; an empty vector (pCMV6) was used as a transfection control. Following transfection, cells were switched to 1% FBS overnight and then treated for four hours with 5 μM T + 1 μM 9cisRA or DMSO vehicle control and RNA was profiled as in (A) *Cyclophilin A* was used as a control for all qRT-PCR reactions. Panels A and B represent results from three independent experiments. Error bars represent SEM. Significance is determined using the two-tailed Student's t-test (*, P < 0.05, **, P<0.01, ***, P<0.001). Error bars represent SD of three technical replicates.
(TIF)

S2 Fig Ligand stimulated induction of Abcg1, Apoe, LPL and Srebp1c are unaffected by PRMT2 levels. BMDMs from wild type and *Prmt2* ^-/-^ mice were cultured in normal glucose, placed in 1% FBS overnight, and treated for four hours with 5 μM T+ 1 μM 9cisRA or DMSO vehicle control and steady state RNA transcripts of *Srebp1c*, *Lpl* and *ApoE* were profiled relative to *Cyclophilin A* by qRT-PCR. This represents results from an individual experiment performed in duplicate. Error bars represent the range of the means.
(TIF)

S3 Fig Individual LXR/RXR ligand induction of Abca1 is less affected by PRMT2 levels. BMDMs from wild type and *Prmt2* ^-/-^ mice were cultured in normal glucose, placed in 1% FBS overnight and treated for four hours with DMSO, 5 μM T, 1 μM 9cisRA or both and expression of *Abca1* relative to *Cyclophilin A* was determined by qRT-PCR. This experiment represents a single experiment performed in triplicate. Error bars represent SD of three technical replicates.
(TIF)

## References

[1] Alexander CM, Landsman PB, Teutsch SM, Haffner SM, Third National H, Nutrition Examination S, et al NCEP-defined metabolic syndrome, diabetes, and prevalence of coronary heart disease among NHANES III participants age 50 years and older. Diabetes. 2003;52(5):1210–4. Epub 2003/04/30. . 12716754 10.2337/diabetes.52.5.1210

[2] Chait A, Bornfeldt KE. Diabetes and atherosclerosis: is there a role for hyperglycemia? Journal of lipid research. 2009;50 Suppl:S335–9. 10.1194/jlr.R800059-JLR200 19029122 PMC2674740

[3] Dorman JS, Tajima N, LaPorte RE, Becker DJ, Cruickshanks KJ, Wagener DK, et al The Pittsburgh Insulin-Dependent Diabetes Mellitus (IDDM) Morbidity and Mortality Study: case-control analyses of risk factors for mortality. Diabetes care. 1985;8 Suppl 1:54–60. Epub 1985/09/01. . 4053954 10.2337/diacare.8.1.s54

[4] Turner RC, Millns H, Neil HA, Stratton IM, Manley SE, Matthews DR, et al Risk factors for coronary artery disease in non-insulin dependent diabetes mellitus: United Kingdom Prospective Diabetes Study (UKPDS: 23). Bmj. 1998;316(7134):823–8. Epub 1998/04/29. 9549452 10.1136/bmj.316.7134.823 PMC28484

[5] Moreno PR, Murcia AM, Palacios IF, Leon MN, Bernardi VH, Fuster V, et al Coronary composition and macrophage infiltration in atherectomy specimens from patients with diabetes mellitus. Circulation. 2000;102(18):2180–4. Epub 2000/11/01. . 11056089 10.1161/01.cir.102.18.2180

[6] Fox CS, Coady S, Sorlie PD, Levy D, Meigs JB, D'Agostino RB Sr., et al Trends in cardiovascular complications of diabetes. Jama. 2004;292(20):2495–9. Epub 2004/11/25. 10.1001/jama.292.20.2495 . 15562129

[7] Kannel WB, McGee DL. Diabetes and glucose tolerance as risk factors for cardiovascular disease: the Framingham study. Diabetes care. 1979;2(2):120–6. Epub 1979/03/01. . 520114 10.2337/diacare.2.2.120

[8] Mazzone T, Chait A, Plutzky J. Cardiovascular disease risk in type 2 diabetes mellitus: insights from mechanistic studies. Lancet. 2008;371(9626):1800–9. Epub 2008/05/27. 10.1016/S0140-6736(08)60768-0 18502305 PMC2774464

[9] Breslow JL. Mouse models of atherosclerosis. Science. 1996;272(5262):685–8. Epub 1996/05/03. . 8614828 10.1126/science.272.5262.685

[10] Feig JE, Parathath S, Rong JX, Mick SL, Vengrenyuk Y, Grauer L, et al Reversal of hyperlipidemia with a genetic switch favorably affects the content and inflammatory state of macrophages in atherosclerotic plaques. Circulation. 2011;123(9):989–98. Epub 2011/02/23. 10.1161/CIRCULATIONAHA.110.984146 21339485 PMC3131163

[11] Smithies O, Maeda N. Gene targeting approaches to complex genetic diseases: atherosclerosis and essential hypertension. Proceedings of the National Academy of Sciences of the United States of America. 1995;92(12):5266–72. Epub 1995/06/06. 7777495 10.1073/pnas.92.12.5266 PMC41675

[12] Feig JE, Pineda-Torra I, Sanson M, Bradley MN, Vengrenyuk Y, Bogunovic D, et al LXR promotes the maximal egress of monocyte-derived cells from mouse aortic plaques during atherosclerosis regression. The Journal of clinical investigation. 2010;120(12):4415–24. Epub 2010/11/03. 10.1172/JCI38911 21041949 PMC2993578

[13] Repa JJ, Liang G, Ou J, Bashmakov Y, Lobaccaro JM, Shimomura I, et al Regulation of mouse sterol regulatory element-binding protein-1c gene (SREBP-1c) by oxysterol receptors, LXRalpha and LXRbeta. Genes & development. 2000;14(22):2819–30. Epub 2000/11/23. 11090130 10.1101/gad.844900 PMC317055

[14] Schwartz K, Lawn RM, Wade DP. ABC1 gene expression and ApoA-I-mediated cholesterol efflux are regulated by LXR. Biochemical and biophysical research communications. 2000;274(3):794–802. Epub 2000/08/05. 10.1006/bbrc.2000.3243 . 10924356

[15] Gabbi C, Warner M, Gustafsson JA. Action mechanisms of Liver X Receptors. Biochemical and biophysical research communications. 2014;446(3):647–50. Epub 2013/12/05. 10.1016/j.bbrc.2013.11.077 . 24300092

[16] Janowski BA, Willy PJ, Devi TR, Falck JR, Mangelsdorf DJ. An oxysterol signalling pathway mediated by the nuclear receptor LXR alpha. Nature. 1996;383(6602):728–31. Epub 1996/10/24. 10.1038/383728a0 . 8878485

[17] Teboul M, Enmark E, Li Q, Wikstrom AC, Pelto-Huikko M, Gustafsson JA. OR-1, a member of the nuclear receptor superfamily that interacts with the 9-cis-retinoic acid receptor. Proceedings of the National Academy of Sciences of the United States of America. 1995;92(6):2096–100. Epub 1995/03/14. 7892230 10.1073/pnas.92.6.2096 PMC42430

[18] Willy PJ, Umesono K, Ong ES, Evans RM, Heyman RA, Mangelsdorf DJ. LXR, a nuclear receptor that defines a distinct retinoid response pathway. Genes & development. 1995;9(9):1033–45. Epub 1995/05/01. . 7744246 10.1101/gad.9.9.1033

[19] Parathath S, Grauer L, Huang LS, Sanson M, Distel E, Goldberg IJ, et al Diabetes adversely affects macrophages during atherosclerotic plaque regression in mice. Diabetes. 2011;60(6):1759–69. Epub 2011/05/13. 10.2337/db10-0778 21562077 PMC3114401

[20] Chawla A, Boisvert WA, Lee CH, Laffitte BA, Barak Y, Joseph SB, et al A PPAR gamma-LXR-ABCA1 pathway in macrophages is involved in cholesterol efflux and atherogenesis. Molecular cell. 2001;7(1):161–71. Epub 2001/02/15. . 11172721 10.1016/s1097-2765(01)00164-2

[21] Costet P, Luo Y, Wang N, Tall AR. Sterol-dependent transactivation of the ABC1 promoter by the liver X receptor/retinoid X receptor. The Journal of biological chemistry. 2000;275(36):28240–5. Epub 2000/06/20. 10.1074/jbc.M003337200 . 10858438

[22] Schultz JR, Tu H, Luk A, Repa JJ, Medina JC, Li L, et al Role of LXRs in control of lipogenesis. Genes & development. 2000;14(22):2831–8. Epub 2000/11/23. 11090131 10.1101/gad.850400 PMC317060

[23] Jenuwein T, Allis CD. Translating the histone code. Science. 2001;293(5532):1074–80. Epub 2001/08/11. 10.1126/science.1063127 . 11498575

[24] Lachner M, O'Sullivan RJ, Jenuwein T. An epigenetic road map for histone lysine methylation. Journal of cell science. 2003;116(Pt 11):2117–24. Epub 2003/05/06. 10.1242/jcs.00493 . 12730288

[25] Like AA, Rossini AA. Streptozotocin-induced pancreatic insulitis: new model of diabetes mellitus. Science. 1976;193(4251):415–7. Epub 1976/07/30. . 180605 10.1126/science.180605

[26] Meyer R, Wolf SS, Obendorf M. PRMT2, a member of the protein arginine methyltransferase family, is a coactivator of the androgen receptor. The Journal of steroid biochemistry and molecular biology. 2007;107(1–2):1–14. Epub 2007/06/26. 10.1016/j.jsbmb.2007.05.006 . 17587566

[27] Qi C, Chang J, Zhu Y, Yeldandi AV, Rao SM, Zhu YJ. Identification of protein arginine methyltransferase 2 as a coactivator for estrogen receptor alpha. The Journal of biological chemistry. 2002;277(32):28624–30. Epub 2002/06/01. 10.1074/jbc.M201053200 . 12039952

[28] Torra IP, Ismaili N, Feig JE, Xu CF, Cavasotto C, Pancratov R, et al Phosphorylation of liver X receptor alpha selectively regulates target gene expression in macrophages. Molecular and cellular biology. 2008;28(8):2626–36. Epub 2008/02/06. 10.1128/MCB.01575-07 18250151 PMC2293109

[29] Murphy AJ, Akhtari M, Tolani S, Pagler T, Bijl N, Kuo CL, et al ApoE regulates hematopoietic stem cell proliferation, monocytosis, and monocyte accumulation in atherosclerotic lesions in mice. The Journal of clinical investigation. 2011;121(10):4138–49. Epub 2011/10/05. 10.1172/JCI57559 21968112 PMC3195472

[30] Yoshimoto T, Boehm M, Olive M, Crook MF, San H, Langenickel T, et al The arginine methyltransferase PRMT2 binds RB and regulates E2F function. Experimental cell research. 2006;312(11):2040–53. Epub 2006/04/18. 10.1016/j.yexcr.2006.03.001 . 16616919

[31] Reddy TE, Pauli F, Sprouse RO, Neff NF, Newberry KM, Garabedian MJ, et al Genomic determination of the glucocorticoid response reveals unexpected mechanisms of gene regulation. Genome research. 2009;19(12):2163–71. Epub 2009/10/06. 10.1101/gr.097022.109 19801529 PMC2792167

[32] Sparrow CP, Baffic J, Lam MH, Lund EG, Adams AD, Fu X, et al A potent synthetic LXR agonist is more effective than cholesterol loading at inducing ABCA1 mRNA and stimulating cholesterol efflux. The Journal of biological chemistry. 2002;277(12):10021–7. Epub 2002/01/16. 10.1074/jbc.M108225200 . 11790770

[33] Brasacchio D, Okabe J, Tikellis C, Balcerczyk A, George P, Baker EK, et al Hyperglycemia induces a dynamic cooperativity of histone methylase and demethylase enzymes associated with gene-activating epigenetic marks that coexist on the lysine tail. Diabetes. 2009;58(5):1229–36. Epub 2009/02/12. 10.2337/db08-1666 19208907 PMC2671038

[34] El-Osta A, Brasacchio D, Yao D, Pocai A, Jones PL, Roeder RG, et al Transient high glucose causes persistent epigenetic changes and altered gene expression during subsequent normoglycemia. The Journal of experimental medicine. 2008;205(10):2409–17. Epub 2008/09/24. 10.1084/jem.20081188 18809715 PMC2556800

[35] Bedford MT, Clarke SG. Protein arginine methylation in mammals: who, what, and why. Molecular cell. 2009;33(1):1–13. Epub 2009/01/20. 10.1016/j.molcel.2008.12.013 19150423 PMC3372459

[36] Ziller MJ, Gu H, Muller F, Donaghey J, Tsai LT, Kohlbacher O, et al Charting a dynamic DNA methylation landscape of the human genome. Nature. 2013;500(7463):477–81. Epub 2013/08/09. 10.1038/nature12433 23925113 PMC3821869

[37] Yang Y, Bedford MT. Protein arginine methyltransferases and cancer. Nature reviews Cancer. 2013;13(1):37–50. Epub 2012/12/14. 10.1038/nrc3409 . 23235912

[38] Lakowski TM, Frankel A. Kinetic analysis of human protein arginine N-methyltransferase 2: formation of monomethyl- and asymmetric dimethyl-arginine residues on histone H4. The Biochemical journal. 2009;421(2):253–61. Epub 2009/05/02. 10.1042/BJ20090268 . 19405910

[39] Le DD, Fujimori DG. Protein and nucleic acid methylating enzymes: mechanisms and regulation. Current opinion in chemical biology. 2012;16(5–6):507–15. Epub 2012/10/23. 10.1016/j.cbpa.2012.09.014 23085277 PMC3545634

[40] Moore KJ, Sheedy FJ, Fisher EA. Macrophages in atherosclerosis: a dynamic balance. Nature reviews Immunology. 2013;13(10):709–21. Epub 2013/09/03. 10.1038/nri3520 23995626 PMC4357520

[41] Miao F, Chen Z, Zhang L, Wang J, Gao H, Wu X, et al RNA-sequencing analysis of high glucose-treated monocytes reveals novel transcriptome signatures and associated epigenetic profiles. Physiological genomics. 2013;45(7):287–99. Epub 2013/02/07. 10.1152/physiolgenomics.00001.2013 23386205 PMC3633422

[42] Barrero MJ, Malik S. Two functional modes of a nuclear receptor-recruited arginine methyltransferase in transcriptional activation. Molecular cell. 2006;24(2):233–43. Epub 2006/10/21. 10.1016/j.molcel.2006.09.020 17052457 PMC1647399

[43] Le Goff W, Zheng P, Brubaker G, Smith JD. Identification of the cAMP-responsive enhancer of the murine ABCA1 gene: requirement for CREB1 and STAT3/4 elements. Arteriosclerosis, thrombosis, and vascular biology. 2006;26(3):527–33. Epub 2005/12/24. 10.1161/01.ATV.0000201042.00725.84 . 16373613

[44] Jansson D, Ng AC, Fu A, Depatie C, Al Azzabi M, Screaton RA. Glucose controls CREB activity in islet cells via regulated phosphorylation of TORC2. Proceedings of the National Academy of Sciences of the United States of America. 2008;105(29):10161–6. Epub 2008/07/16. 10.1073/pnas.0800796105 18626018 PMC2481316

[45] Jakobsson T, Venteclef N, Toresson G, Damdimopoulos AE, Ehrlund A, Lou X, et al GPS2 is required for cholesterol efflux by triggering histone demethylation, LXR recruitment, and coregulator assembly at the ABCG1 locus. Molecular cell. 2009;34(4):510–8. Epub 2009/06/02. 10.1016/j.molcel.2009.05.006 . 19481530

[46] Uhlen M, Oksvold P, Fagerberg L, Lundberg E, Jonasson K, Forsberg M, et al Towards a knowledge-based Human Protein Atlas. Nature biotechnology. 2010;28(12):1248–50. Epub 2010/12/09. 10.1038/nbt1210-1248 . 21139605

[47] Gerstein MB, Kundaje A, Hariharan M, Landt SG, Yan KK, Cheng C, et al Architecture of the human regulatory network derived from ENCODE data. Nature. 2012;489(7414):91–100. Epub 2012/09/08. 10.1038/nature11245 22955619 PMC4154057

[48] Iwasaki H, Kovacic JC, Olive M, Beers JK, Yoshimoto T, Crook MF, et al Disruption of protein arginine N-methyltransferase 2 regulates leptin signaling and produces leanness in vivo through loss of STAT3 methylation. Circulation research. 2010;107(8):992–1001. Epub 2010/08/28. 10.1161/CIRCRESAHA.110.225326 20798359 PMC2997704

