# Supplementary figures and images for: LXR-Mediated ABCA1 Expression and Function Are Modulated by High Glucose and PRMT2
Source: PLoS One. 2015 Aug 19;10(8):e0135218. doi: 10.1371/journal.pone.0135218 (PMC4545936; doi:10.1371/journal.pone.0135218)

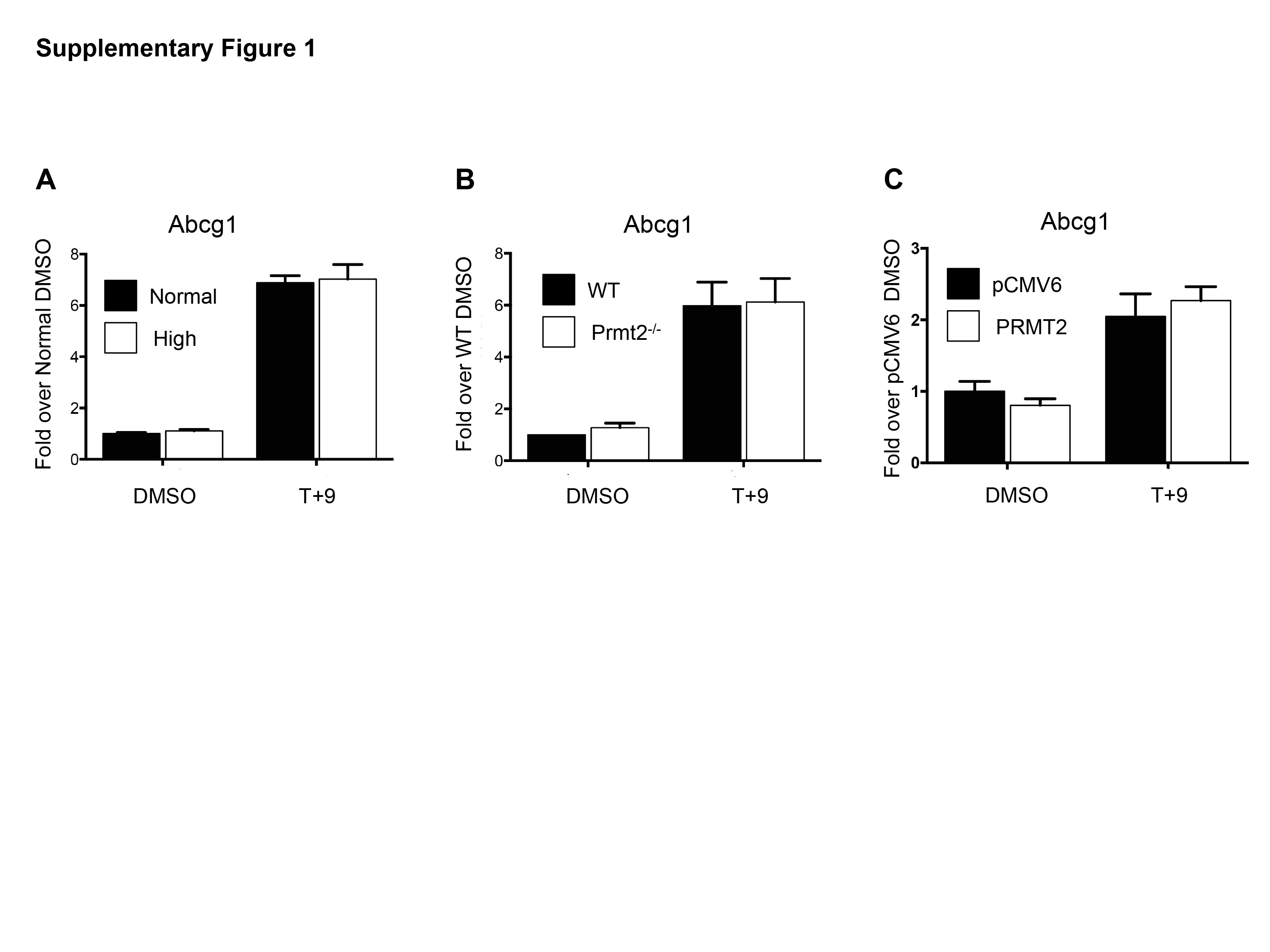

Supplement: S1 Fig — (A) Bone marrow cells from C57BL/6 mice were differentiated into macrophages under high glucose (25 mM D-glucose) or normal glucose (5.5 mM D-glucose + 19.5 mM L-glucose). Prior to treatments, macrophages were cultured in 1% FBS overnight and then treated for four hours with 5 μM T + 1 μM 9cisRA or DMSO vehicle control and steady state RNA transcripts of Abcg1 were profiled using qRT-PCR. (B) BMDMs from wild type and Prmt2 -/- mice were cultured and treated as in (A). (C) Myc-DDK tagged PRMT2 was transfected into RAW WT macrophages; an empty vector (pCMV6) was used as a transfection control. Following transfection, cells were switched to 1% FBS overnight and then treated for four hours with 5 μM T + 1 μM 9cisRA or DMSO vehicle control and RNA was profiled as in (A) Cyclophilin A was used as a control for all qRT-PCR reactions. Panels A and B represent results from three independent experiments. Error bars represent SEM. Significance is determined using the two-tailed Student's t-test (*, P < 0.05, **, P<0.01, ***, P<0.001). Error bars represent SD of three technical replicates. (TIF) [file pone.0135218.s001.tif]

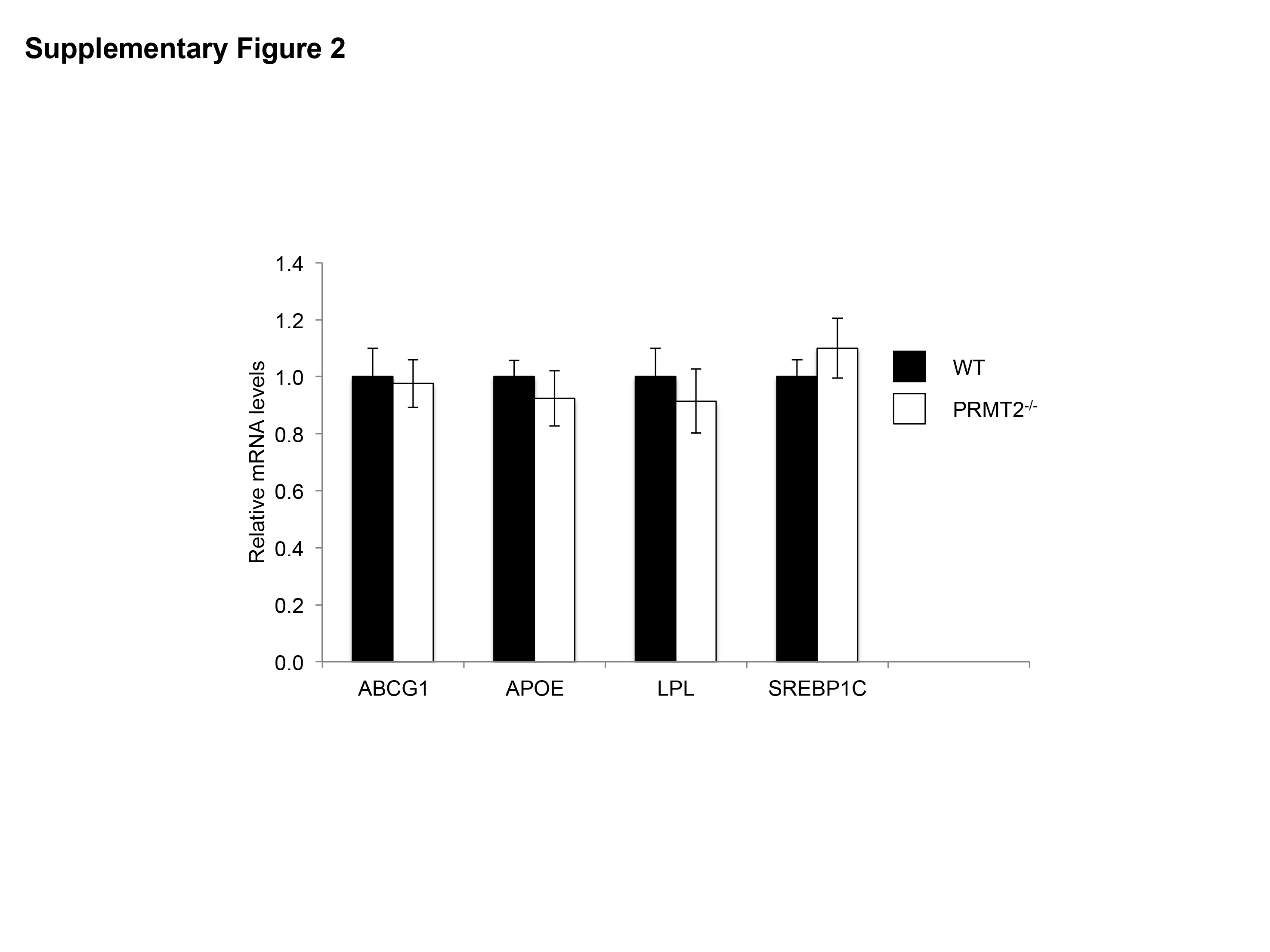

Supplement: S2 Fig — BMDMs from wild type and Prmt2 -/- mice were cultured in normal glucose, placed in 1% FBS overnight, and treated for four hours with 5 μM T+ 1 μM 9cisRA or DMSO vehicle control and steady state RNA transcripts of Srebp1c, Lpl and ApoE were profiled relative to Cyclophilin A by qRT-PCR. This represents results from an individual experiment performed in duplicate. Error bars represent the range of the means. (TIF) [file pone.0135218.s002.tif]

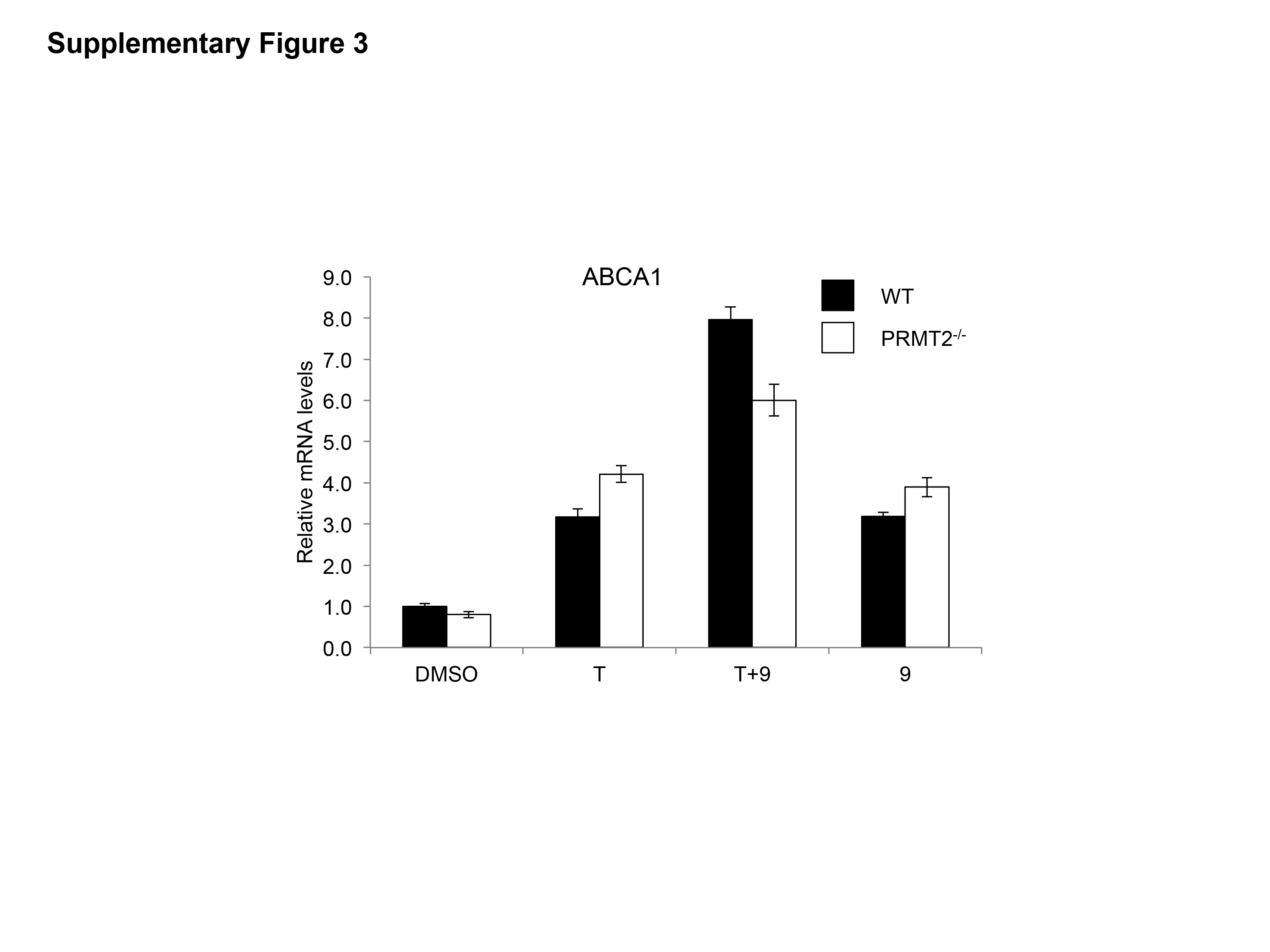

Supplement: S3 Fig — BMDMs from wild type and Prmt2 -/- mice were cultured in normal glucose, placed in 1% FBS overnight and treated for four hours with DMSO, 5 μM T, 1 μM 9cisRA or both and expression of Abca1 relative to Cyclophilin A was determined by qRT-PCR. This experiment represents a single experiment performed in triplicate. Error bars represent SD of three technical replicates. (TIF) [file pone.0135218.s003.tif]
